# Hemi-mandibulectomy without bony reconstruction: A case report of mandibular metastasis from a silent differentiated papillary thyroid cancer

**DOI:** 10.1016/j.amsu.2022.103334

**Published:** 2022-02-04

**Authors:** Artanto Wahyono, Ery Kus Dwianingsih, Widya Surya Avanti, Roby Cahyono, Rosa Mandasari, Sumadi Lukman Anwar

**Affiliations:** a Division of Surgical Oncology - Department of Surgery, Dr Sardjito Hospital / Faculty of Medicine, Public Health, and Nursing, Universitas Gadjah Mada, Yogyakarta, 55281, Indonesia; b Department of Anatomical Pathology, Dr Sardjito Hospital / Faculty of Medicine, Public Health, and Nursing, Universitas Gadjah Mada, Yogyakarta, 55281, Indonesia; c Department of Radiology, Dr Sardjito Hospital / Faculty of Medicine, Public Health, and Nursing, Universitas Gadjah Mada, Yogyakarta, 55281, Indonesia

**Keywords:** Thyroid cancer, Jaw metastases, Hemi-mandibulectomy without reconstruction, CT-scan, Computed Tomography-scan, SCARE, Surgical Case Report, TSH, Thyroid Stimulating Hormone

## Abstract

**Introduction:**

Distant spread to the jaw is a rare metastatic manifestation from papillary thyroid cancer. Complete resection of tumor extension in the facial and oral regions requires consideration to compromise mastication functioning and facial aesthetics. Current advances in the microvascular surgery have facilitated excellent restoration of patient's functioning. Inadequate expertise, facility, longer surgery time and inpatient care, and healthcare insurance disbursement are common challenges in developing countries to perform microvascular surgery.

**Case presentation:**

A 54-year female presented in an oncology clinic with a rapid progressive lump in the jaw without inflammatory signs. CT-scan revealed a 5.9x5.3 × 5cm lesion with osteo-destruction in the left mandible body. Biopsy was performed indicating a papillary adenocarcinoma invasion. Neck sonography showed hypoechoic nodule with regular border in the thyroid lobes. Fine-needle aspiration biopsy revealed benign follicular cells with Bethesda class II. Total thyroidectomy with frozen section and left hemi-mandibulectomy without bony reconstruction were then performed. Histopathological examination showed papillary thyroid cancer with follicular variant in the thyroid and mandible lesion. Thyroid ablation, TSH suppression, and chewing rehabilitation programs were accomplished by the patient.

**Discussion:**

Partial mandibulectomy without bony reconstruction might be an option for selected patients with careful consideration from multidisciplinary team members in which extensive surgery with immediate bony reconstruction is not possible.

## Highlights

• Mandibulectomy without reconstruction can be performed for selected cases.
• Metastasis to the mandible from differentiated thyroid cancer is rare.
• Silent thyroid cancer can be manifested as mandible metastasis.

## 1 Introduction

**Fig. 1:**
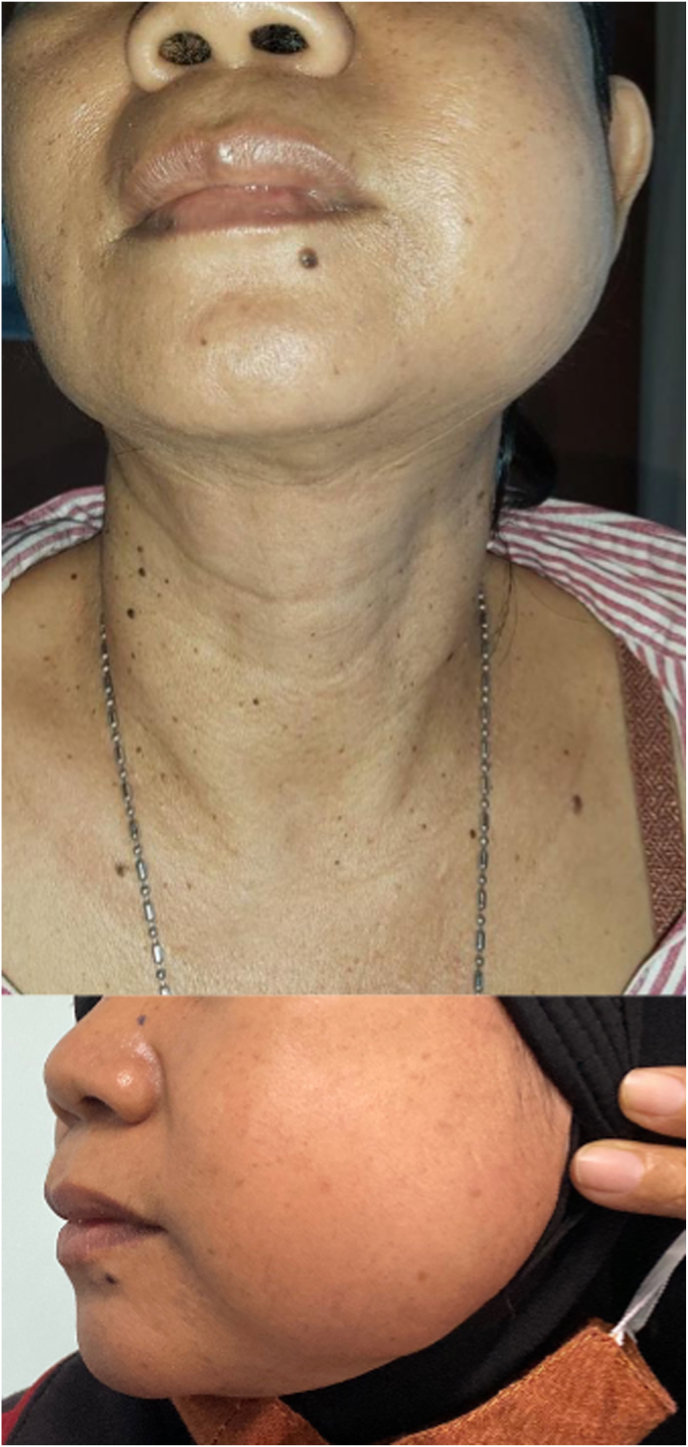
Clinical presentation at diagnosis. A growing lump in the mandible without prominent thyroid lesion.

**Fig. 2:**
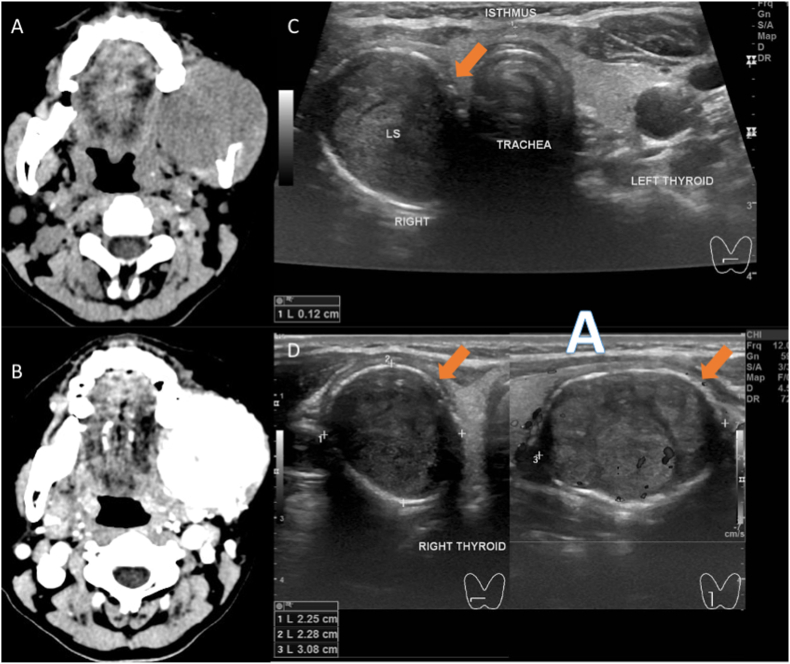
Head computed tomography (CT)-scan showed an intraosseous hypervascularity lesion in the left mandible angle with a size of 5.9x5.3 × 5 cm (A, plain scanning and B after contrast). Neck ultrasonography revealed hypoechoic lesion of 1.97x2.31 × 2.35 cm in the right lobe irregular borders and intralesional vascularization (C and D).

**Fig. 3:**
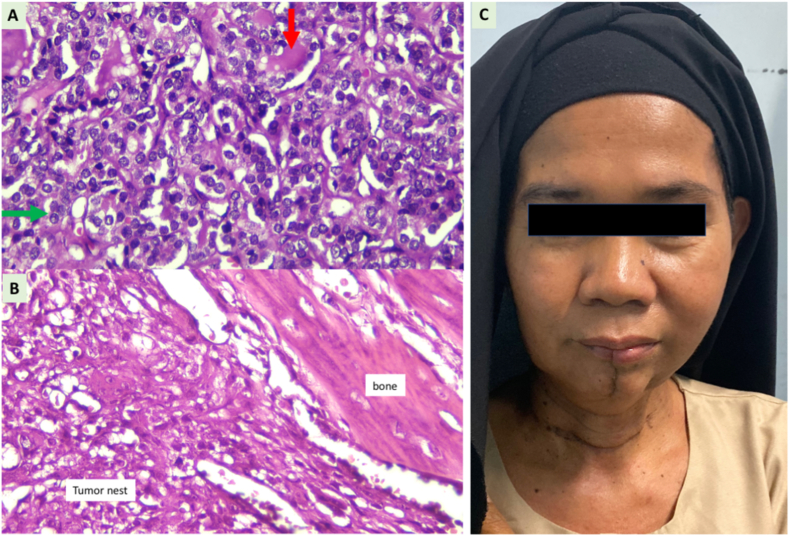
Total thyroidectomy and hemi-mandibulectomy without bony reconstruction were performed. Histopathology examination revealed a follicular variant of thyroid cancer (A) and infiltration of papillary cancer in the mandible bone (B). Tumor is densely arranged in follicular pattern with some lumens are filled with colloid mass (red arrow, (A)). The nuclear features are round to oval, overcrowding, overlapping, and some with pseudonuclear inclusion (green arrow (A). The patient expressed acceptable cosmesis and mastication function (C). (For interpretation of the references to colour in this figure legend, the reader is referred to the Web version of this article.)

Papillary thyroid carcinoma is the most common subtype of well-differentiated thyroid cancer with tendency of favorable clinical course since 98% of patients survive after 10 years [1]. Distant metastasis from papillary thyroid cancer outside the neck area is rare occurring only in 1–3% of patients [2]. The distant spread usually occurs 10–30 years after initial diagnosis [2]. Bone metastasis to the jaw only accounts for 1% of malignancy commonly from oral origins [3]. The tumor cells spread to the jaw either through lymphatic vessels or bloodstream [4]. The posterior part of the mandible is the most commonly affected because of the higher content of vasculature and sinusoidal spaces that facilitate tumor inoculation [4]. Distant spread to the mandible is often viewed as a late-stage manifestation of cancer [3] (see Fig. 1, Fig. 2, Fig. 3).

Around 5% of all distant metastases from differentiated thyroid cancer are found in the oral cavity and most of them are from follicular thyroid cancer [4]. Because the mandible plays a vital role in mastication and facial form, any surgery procedure in the jaw might adversely affect aesthetic and functions [5]. Complete or partial mandibulectomy might also cause sensory and face deformity [6]. In addition, dynamic interactions of the maxillae and mandibulae during the chewing process can be largely distorted after partial or hemi-mandibulectomy [5]. Coordination between the hard and soft palate during food bolus placement can also be deregulated after mandibulectomy [5]. Grinding, trituration, and ability to manipulate food bolus will also be affected particularly after hemi or partial mandibulectomy [5].

The most common procedure for reconstruction after a large mandibulectomy is free fibular flap to improve facial construction, mastication, and the swallowing process [7]. The advances in the technique of microvascular surgery have significantly improved the functional and aesthetic outcomes [7]. However, reconstructive surgery also poses some risks of complications including implant exposure, graft failure, flap necrosis, and face deformation [8]. Extended operation time potentially causes postoperative complications and consumes higher healthcare resources [8]. Therefore, reconstructive surgery should be carefully performed in certain cases after multidisciplinary team discussion involving consideration of patient's needs and expectations [5,7]. In this report, we present an alternative option of hemi-mandibulectomy without reconstruction in a patient with a mandibular metastatic lesion from a silent papillary thyroid cancer following the guidelines of SCARE 2020 recommendations [9].

## 2 Case presentation

A 54-year-old woman had complained about a rapid progressive lump in the jaw for the past one year. She expressed there had been an alteration of sensation in the lower left teeth, slight malocclusion, and a growing pain since the past month. Food chewing and articulation were interrupted due to the lesion. A personal medical history of previous malignancy, radiotherapy exposure, allergy, surgery, and diabetes were denied. Physical examination revealed 5-cm diffuse hard swelling in the mandible body. Intraoral palpation showed a mandible lesion without protruded ulcerated lesion to the oral mucosa. Biopsy in the mandible lesion was performed in the previous hospital and revealed infiltration of papillary carcinoma to the bone. Head CT-scan revealed a 5.9×5.3×5cm amorphic mass in the left mandibular body and bone destruction in the angle. Neck ultrasonography showed a hypoechoic 1x1x1.4cm lesion in the left thyroid lobe, regular border, taller than wide, and increased intra-tumoral vascularization. A 1.97x2.31x2.34 hypoechoic lesion with regular clear border, and intralesional vascularization was observed in the right thyroid lobe. Fine-needle aspiration biopsy in the thyroid nodules revealed a benign follicular nodule with Bethesda System identification of Class II. Because sonography-guided needle biopsy in the thyroid showed benign follicular nodule, right isthmo-thyroidectomy was initially performed. After the frozen section showed malignancy, total thyroidectomy was then performed. Left hemi-mandibulectomy without bony reconstruction was performed afterwards. Histopathology examination showed follicular variants of papillary thyroid carcinoma both in the thyroid lobes and the mandible lesion. The patient was categorized in the high-risk papillary thyroid cancer due to histology with lympho-vascular invasion and manifestation of distant metastases. Thyroid ablation, TSH suppression, and chewing exercises were accomplished by the patient. She expressed an alteration in the ability to place food during mastication. The complaint improved with chewing exercises in the medical rehabilitation program. Four weeks after surgery, she was able to eat soft food. In the one-year follow-up, the patient was able to eat solid to hard food and to chew certain cooked vegetables. Neck sonography evaluation one year after surgery showed no thyroid remnant and TSH was maintained at 0.01 μIU/mL.

## 3 Clinical discussion

Papillary thyroid carcinoma has been considered as indolent cancer with lower propensity to metastasize to distant organs. In circumstances of delayed diagnosis or inadequate initial detection and treatment, however, we have reported some cases of papillary thyroid cancer with large bone resorption [10] and gross extrathyroidal extension [11]. Several factors including large tumor size, lymphovascular invasion, and certain aggressive variants are associated with distant metastases [2,12]. Extrathyroidal extension, and positive margins are associated with risks of distant metastases [13]. Early detection of mandible metastasis from papillary thyroid cancer is often difficult particularly if the primary tumor is relatively small and is presented without mechanical obstruction [[14], [15], [16]]. Several reports showed that most mandible metastasis were reported among men and in the posterior mandible regions [4,17].

Patients’ complaints of mandible metastasis were gradual swelling with pain, tooth instability, and pricking sensation in the face. Our patient presented in the oncology clinic because of jaw swelling with pain. If the mental nerve is affected by the metastasis, patients usually feel paresthesia or numb-chin syndrome [4]. Around 14% of patients with mandible metastases showed tooth instability [18]. In this case, the patient did not specifically complain about goiter nor the associated signs of mechanical compression.

Distant metastases from thyroid cancer commonly arise from follicular thyroid carcinoma with specific characteristics of Hurtle cells, poor differentiation, and vascular invasion [13]. In contrast, papillary thyroid cancer is a slow growing tumor and is often detected incidentally [13]. Distant metastasis from papillary thyroid cancer is very rare and usually occurs after 10–20 years follow-up [13,17]. The most common sites for bone metastasis from thyroid cancer are the vertebrae, sternum, and pelvis [14,17]. In the radiological imaging, metastatic bone lesions from thyroid cancer are usually displayed as irregular radiolucent lesions [3]. If papillary thyroid carcinoma shows intermediate clinical characteristics between papillary and follicular carcinomas, it can be considered as a follicular variant of papillary thyroid cancer if the histopathology shows a follicular pattern with the ground-glass nuclear features [19]. In addition, specific characteristics of follicular variants of papillary thyroid carcinoma include the presence of distant metastasis without lymph node infiltration [19] which was also found in our presented case. Both primary cancer and the metastatic lesion in the mandible in our case showed a follicular variant of papillary thyroid cancer. However, follicular variants of papillary thyroid carcinoma should not be confused with follicular thyroid cancer since it usually grows slowly and is not included in the high-risk variants [19]. In the imaging, the follicular variant usually shows vascular prominence due to the arterial malformation in the tumor growth [20] which was also seen in our patient with a rapid growing mandible lump and hyper-vascularization.

Distant metastases to the oral cavity from thyroid carcinoma usually affect older female patients. Involvement of the mandible is usually associated with greater vasculature invasion in the primary tumor [21]. The mandibula has a vital function in the opening and closing of the mouth during mastication [22]. In addition, the masticator muscles consist of masseter, temporalis, lateral and medial pterygoid muscles that attach to the mandible [22]. Therefore, hemi-mandibulectomy can cause significant mastication impairment. The mandible and masticator muscles also form support for the lower face and partial loss of the structure will have aesthetic consequences [22]. Current guidelines suggest reconstruction of mandible defects with bone and soft tissue transfer with dental rehabilitation. Fibular flap is the most commonly used method for reconstruction after hemi-mandibulectomy or large mandible defects. However, complex surgery and longer state of anesthesia have higher risks of complications including failure of reconstruction that might cause malocclusion, mastication problems, and temporo-mandibular joint pain [23]. Patients with comorbidities and elderly have higher risks of complications. In a study of 54 patients with free fibular flap, around 50% of patients reported functional disturbances and poor aesthetic outcomes [24]. With the availability of new techniques including virtual surgical planning, the reconstructive surgery has been significantly improved in the accuracy, surgical time efficiency, and acceptance of aesthetic outcomes [25]. Reconstruction using titanium mandibular plate is an option with a relatively higher rate of complications. Clinical determinants including older age, poor performance status, cigarette smoking, and adjuvant radiotherapy increase risks of complications and dissatisfaction [26].

After careful discussion with the patient, hemi-mandibulectomy without bony reconstruction was performed considering that potential adjuvant radiotherapy might be required. Mandible resection without additional reconstruction is a reasonable option in selective cases in which free bony or tissue reconstruction is difficult particularly in the presence of multiple comorbidities in elderly individuals, a need of adjuvant radiotherapy, and heavy smoking history. However, dental rehabilitation cannot be performed if bony reconstruction is not performed after mandible resection. Partial mandibulectomy without bony reconstruction has also been associated with acceptable chewing and mouth opening functions. In addition, mandibulectomy without reconstruction also results in cosmetic satisfaction, less pain, and less medical complications after radiotherapy [8]. In addition, mandible reconstruction might require several months to achieve acceptable functional and cosmetic results [5]. Reassurance and counseling are required during the recovery [5]. Therefore, mandibulectomy without reconstruction needs to be carefully selected especially for patients who promptly need adjuvant radiotherapy.

## 4 Conclusions

Although reported as a very rare event, distant metastasis to the mandible can become the first manifestation of a silent papillary thyroid cancer. Hemi-mandibulectomy without bony reconstruction might be an acceptable option for patients with certain conditions and comorbidities that prevent them from further risk of complications. Partial mandibulectomy without reconstruction also offers fulfilling aesthetic considerations and mastication function.

## Sources of funding

This report did not receive specific funding.

## Ethics approval

Not applicable.

## Consent for publication

Written informed consent was obtained from the patient for reporting the case and displaying the relevant images. De-identification of images and related materials were used in this manuscript. A copy of the written informed consent is available for review by the Editor-in-Chief of this journal on request.

## Authors’ contributions

SLA, RC, RM, and AW conceptualized the report, produce the imaging, and finalized the manuscript. EKD provided and gave expertise in the tumor histopathology. WSA gave expertise in the imaging. All authors read and approved the final manuscript.

## Registration of research study

Not applicable.

## Guarantor

SLA.

## Provenance and peer review

Not commissioned, externally peer reviewed.

## Availability of data and materials

The clinical and imaging data supporting the analysis and findings of this study will be available from the corresponding author upon reasonable request.

## Declaration of competing interest

No potential competing interest has been declared from all authors.
